# Triggering RNA Interference by Photoreduction under Red Light Irradiation

**DOI:** 10.3390/molecules28104204

**Published:** 2023-05-20

**Authors:** Jennifer Rühle, Insa Klemt, Andriy Mokhir

**Affiliations:** Department of Chemistry and Pharmacy, Organic Chemistry II, Friedrich-Alexander-University of Erlangen-Nürnberg (FAU), Nikolaus-Fiebiger Str. 10, 91058 Erlangen, Germany; jennifer.ruehle@fau.de

**Keywords:** siRNA prodrug, selective cancer targeting, red light activation, photoreduction, RNAi, azobenzene, promoiety

## Abstract

RNA interference (RNAi) using small interfering RNAs (siRNAs) is a powerful tool to target any protein of interest and is becoming more suitable for in vivo applications due to recent developments in RNA delivery systems. To exploit RNAi for cancer treatment, it is desirable to increase its selectivity, e.g., by a prodrug approach to activate the siRNAs upon external triggering, e.g., by using light. Red light is especially well suited for in vivo applications due to its low toxicity and higher tissue penetration. Known molecular (not nanoparticle-based) red-light-activatable siRNA prodrugs rely on singlet oxygen (^1^O_2_)-mediated chemistry. ^1^O_2_ is highly cytotoxic. Additionally, one of the side products in the activation of the known siRNA prodrugs is anthraquinone, which is also toxic. We herein report on an improved redlight-activatable siRNA prodrug, which does not require ^1^O_2_ for its activation. In fact, the 5′ terminus of the antisense strand is protected with an electron-rich azobenzene promoiety. It is reduced and cleaved upon red light exposure in the presence of Sn(IV)(pyropheophorbide a)dichloride acting as a catalyst and ascorbate as a bulk reducing agent. We confirmed the prodrug activation upon red light irradiation both in cell-free settings and in human ovarian cancer A2780 cells.

## 1. Introduction

**Figure 1:**
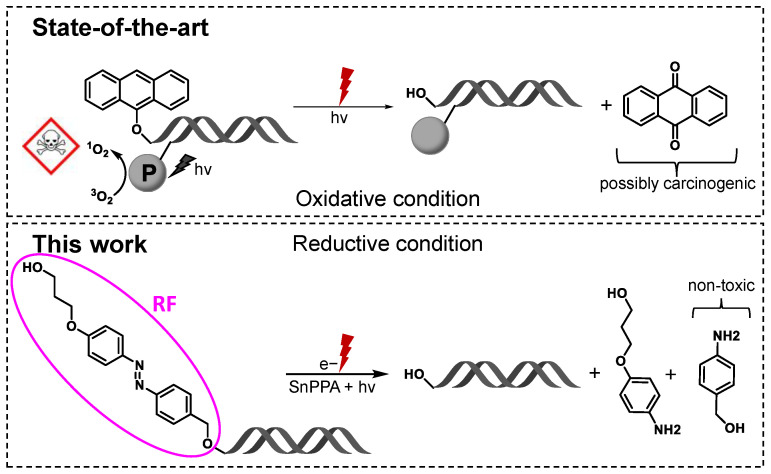
The state of the art in siRNA prodrugs. The 9-anthracenyl promoiety on the guide strand of the siRNA is cleaved by ^1^O_2_ that is photogenerated on the passenger strand upon red light irradiation. The ^1^O_2_, as well as the anthraquinone side product, is potentially harmful to the cells and the surrounding tissue. In this work, we report on an improved siRNA prodrug, which does not require ^1^O_2_ for the activation: it is activated upon red light-induced electron transfer in the presence of photosensitizer SnPPA.

**Scheme 1:**
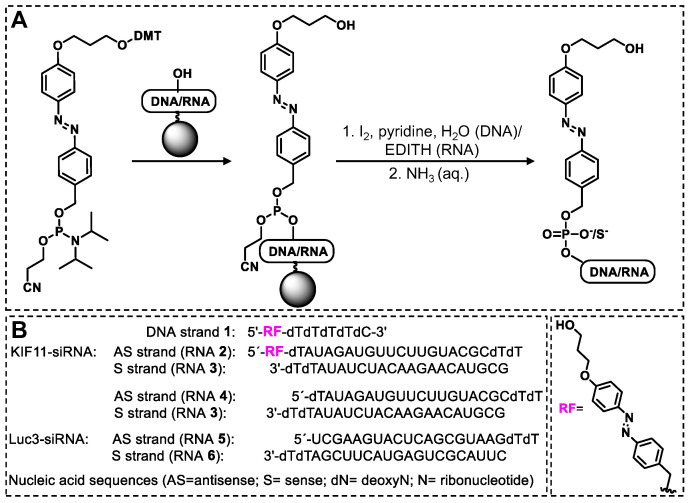
(**A**) Synthesis scheme of DNA **1**/RNA **2** by solid-phase synthesis. While the 5′ P(III) of DNA **1** was oxidized by I_2_, pyridine in H_2_O, the 5′ P(III) of RNA **2** was oxidized with 3-ethoxy-1,2,4-thiazoline-5-one (EDITH), giving rise to the phosphodiester and the phosphorothioate, respectively. (**B**) Structure of promoiety RF and sequences of DNA/RNAs used in this work.

Due to recent developments in in vivo oligonucleotide delivery systems, RNA-based therapeutic approaches are becoming more and more enticing. Hence, alongside, for example, RNA-based vaccines, five silencing RNA (siRNA) drugs have been clinically approved (patisiran, givosiran, lumasiran, inclisiran, and vutrisiran) that act via the mechanism of RNA interference (RNAi) to downregulate specific genes [1,2,3,4,5]. All five siRNA drugs on the market treat metabolic or neurodegenerative diseases. As an alternative application, targeting cancer cells by the downregulation of genes that are essential for mitosis, for example, would be attractive as well. In that way, proteins that are considered undruggable could also be targeted. However, similar to small molecular drug approaches, the mere targeting of mitosis and thus fast-dividing cells, results in dose-limiting side effects concerning non-cancerous cells that rely on fast proliferation, resulting in myelosuppression, cytopenia, hepatotoxicity, etc. One possible solution to increase cancer selectivity is the application of prodrugs that are activated in a specific cancer microenvironment or upon an external trigger. Accordingly, few examples of siRNA prodrugs have been reported [6,7,8]. Following this approach, we have already developed ROS-responsive siRNAs that are activated by elevated H_2_O_2_ concentrations [9], a hallmark of many cancers [10]. In addition, we reported on red-light-responsive siRNAs (Figure 1), in which the passenger strand is modified with a photosensitizer that generates ^1^O_2_ upon red light irradiation. The on-demand-generated ^1^O_2_ cleaves the 9-anthracenyl promoiety on the antisense strand, yielding the active siRNA, and while the activation is efficient, side effects by these harsh oxidative conditions are to be expected, especially for in vivo applications. This can be one of the reasons why this system has never been applied in vivo. In search of alternative promoieties, which are not activated under strong oxidative conditions, we explored azobenzenes. The application of azobenzene triggers in a biological context comes with some challenges, as two distinct processes can be initiated, the first one being photoisomerization and the second one being reduction. The photoisomerization of azobenzenes is in most cases triggered by UV light, which is toxic to cells and does not show significant tissue penetration. Nevertheless, some stable azobenzenes were successfully applied in living cells or in vivo to activate certain processes (e.g., disassembly of complex biomolecules) by photoswitching to the *cis* isomer [11,12,13]. Exploiting the second process, few approaches have also been developed towards reducible azobenzenes, e.g., as sensors of hypoxia [14,15]. For this study, we aimed for a stable azobenzene moiety to avoid the unspecific intracellular reduction of the siRNA prodrug. However, upon an external trigger, the promoiety should be reduced efficiently. We therefore selected electron-rich azobenzene “RF” (Scheme 1), which was shown to be non-cleavable under conditions mimicking the cellular environment [16]. We hoped that this residue would still be photo-reducible in the presence of catalyst Sn(IV)(pyropheophorbide a)dichloride (SnPPA) (Figure 1). We selected SnPPA based on our previous report [16], in which we established that in contrast to free PPA and its complexes (e.g., In(III)(pyropheophorbide a) chloride, InPPA), SnPPA is able to mediate the photoreduction by electron transfer to organic substrates.

In preliminary tests, an RF-DNA model strand (DNA **1**) was synthesized, characterized, and its stability in the presence of various potentially reactive molecules found in the intracellular environment (H_2_O_2_, NaSH, KO_2_, and sodium ascorbate) was confirmed. In contrast, we observed that DNA **1** is indeed activated upon irradiation with red light in the presence of photosensitizer SnPPA and ascorbate as an electron source. Next, the RF-RNA guide strand (RNA **2**) was synthesized and its similarity to DNA **1**′s behavior was confirmed in cell-free settings. Finally, the corresponding siRNA prodrug (RNA **2**/**3**) was assembled. As a gene target, *KIF11* was chosen because it is an essential gene for mitosis that is overexpressed in some cancers, making it a promising target for cancer treatment [17]. However, it is an essential gene expressed in many organs [18], which makes it necessary to activate the inhibition of siRNA specifically in cancer cells to achieve the favorable therapeutic effect. We investigated the RNAi efficiency of the siRNA prodrug in human ovarian carcinoma cells A2780 by reverse-transcription quantitative PCR (RT-qPCR) in the presence and absence of the photosensitizer SnPPA, showing selective activation and good knockdown efficiency upon irradiation with red light.

## 2. Results

### 2.1. Design, Synthesis, and Characterization of DNA ***1*** and RNA ***2***

**Figure 2:**
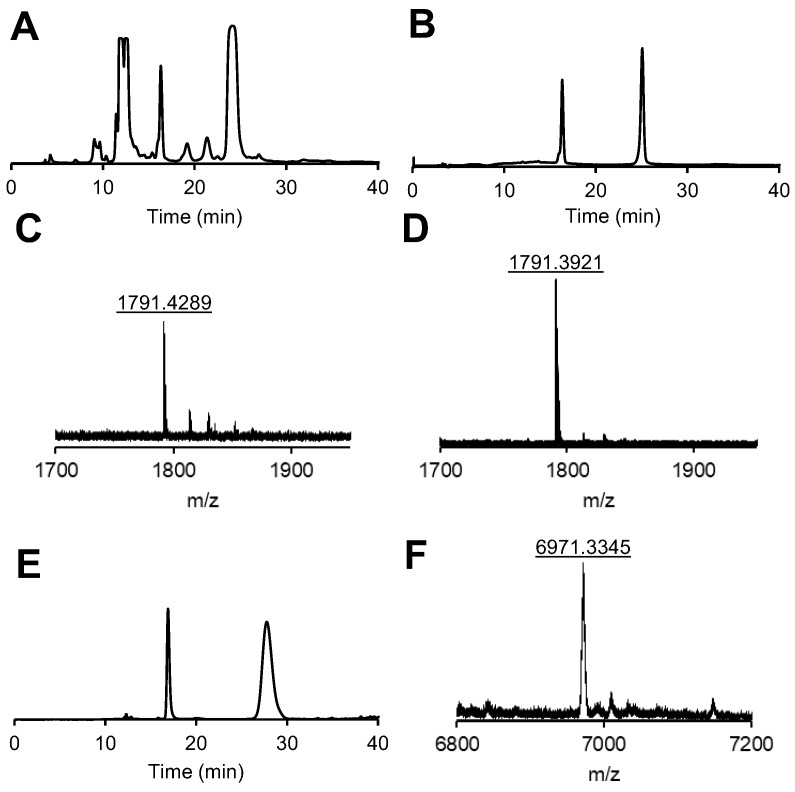
Crude (**A**) and analytical (**B**) HPLC profile of DNA **1** and MALDI-TOF-MS spectra of the peaks in (**B**) eluted after 18 min (**C**) and 25 min (**D**). Analytical HPLC of RNA **2** (**E**) and corresponding MALDI-TOF-MS spectra of the peak eluted after 28 min (**F**). Reversed-phase HPLC was performed with a gradient of solution B (ACN) in solution A (TEAA buffer, 150 mM, 5% ACN, pH 7.4): From 0 to 10 min to 15% B until 15 min; from 15 to 20 min to 20% B until 35 min (DNA **1**) and from 0 to 10 min to 15% B until 20 min, then from 20 to 40 min to 30% B (RNA **2**) by monitoring the absorbance at 260 nm.

The RF-phosphoramidite (Scheme S1) was synthesized as described earlier [16] and applied for the solid-phase synthesis of model strand DNA **1** using commercially available standard DNA phosphoramidites (Scheme 1 and Scheme S2, Supporting Information (further SI)). The analytical HPLC profile of the crude synthesis mixture indicates that the overall yield is higher than 50% and is shown in Figure 2A. The purification of the DNA by reversed-phase chromatography gives rise to the analytically pure product as confirmed by HPLC (Figure 2B). We identified the obtained product by MALDI-TOF-MS. In particular, we observed two peaks eluted after 18 min and 25 min with the identical mass of 1791 Da (Figure 2C,D) that correspond to the expected brutto formula of DNA **1** (calculated for C_65_H_82_N_13_O_37_P_5_ [M − H^+^]^−^: *m*/*z* = 1791 Da). We calibrated MALDI-TOF-MS by using an external standard, which gives rise to an accuracy of determination in *m*/*z* = 0.1% of the detected mass (e.g., for a peak with *m*/*z* = 1791 Da, the expected deviation would be +/− 1.8 Da). Thus, the product is a mixture of two isomers corresponding to the *cis* and the *trans* forms of the azobenzene-DNA.

We prepared the RNA part of the RF-RNA strand **2** (Scheme 1 and Scheme S3) by solid-phase RNA synthesis using commercially available dioxo-thiomorpholine (TC) RNA phosphoramidites according to the manufacturer’s recommendations. We conducted the coupling of the azobenzene phosphoramidite RF analogously to DNA **1** synthesis, except that we performed the oxidation step with 3-ethoxy-1,2,4-thiazoline-5-one (EDITH) to yield the corresponding phosphorothioate. This modification was utilized, as it shows improved stability towards unspecific phosphatases in cells. The characterization (analytical HPLC and MALDI-TOF-MS spectra) is shown in Figure 2E,F. As expected, again two distinct peaks with identical mass were observed (calculated for C_216_H_266_N_71_O_152_P_21_S [M − H^+^]^−^: *m*/*z* = 6970 Da; found: *m*/*z* = 6971 Da), corresponding to the *cis* and the *trans* isomers of RNA **2**. The sense-strand RNA **3**, the unmodified *KIF11* RNA antisense strand RNA **4**, and the RNA strands for a control (scrambled) siRNA without any cellular target (RNA **5** and RNA **6**) were custom-made by Sigma Aldrich (Scheme 1). We annealed the RNA strands via temperature gradient (from 90 °C for 15 min to 22 °C in 45 min) in the presence of high salt concentrations (500 mM NaCl) and ethylenediaminetetraacetic acid (EDTA, 10 mM).

### 2.2. Cis–Trans Isomerization of DNA ***1*** and Its Photoreduction

**Figure 3:**
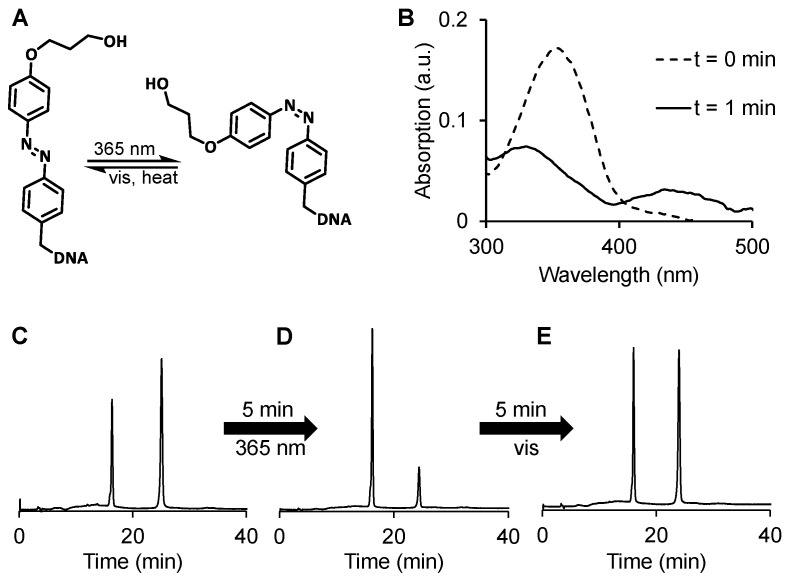
Cis-trans isomerization of DNA **1** (**A**) as observed by UV vis absorbance (**B**) before and after 1 min irradiation at 365 nm. HPLC profile (absorbance at 260 nm) of DNA **1** before (**C**) and after (**D**) 5 min irradiation at 365 nm and after an additional 5 min at room light (**E**), using a gradient of solution B (ACN) in solution A (TEAA buffer, 150 mM, 5% ACN, pH 7.4): From 0 to 10 min to 15% B until 15 min; from 15 to 20 min to 20% B until 35 min.

To investigate the *cis*–*trans* isomerization of DNA **1** (Figure 3A) and to further prove the identity of the two elution peaks in the HPLC profile, a UV-vis spectrum of DNA **1** was recorded before and after irradiation with 365 nm for 1 min (Figure 3B). As expected, the absorbance at 365 nm, which is characteristic of the *trans* isomer, decreases upon irradiation, while the absorbance at 440 nm, characteristic of the *cis* isomer, increases. In the HPLC profile, light irradiation shifts the equilibrium toward the peak that is eluted first (compare Figure 3C,D), indicating that this peak corresponds to the *cis* isomer. Already five minutes after the irradiation, the equilibrium shifts nearly completely back to the initial state (Figure 3E).

**Scheme 2:**
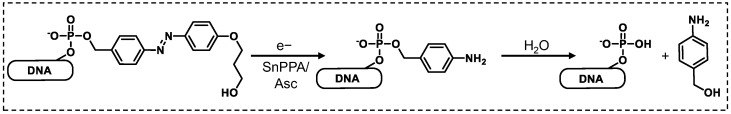
Cleavage mechanism of DNA **1** upon irradiation with red light in the presence of SnPPA and ascorbate (Asc).

**Figure 4:**
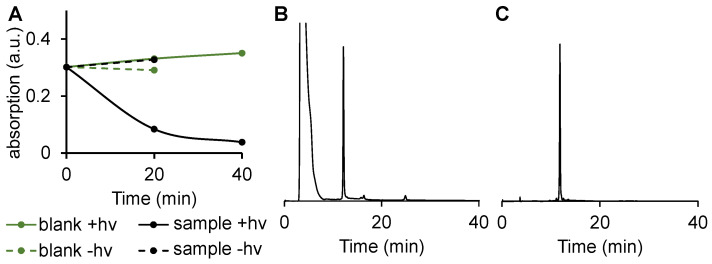
Photoreduction of DNA **1** in PBS (10 mM, 150 mM NaCl, 10 mM sodium ascorbate, pH 7.4) in the presence (“sample”) or absence (“blank”) of SnPPA (4 µM). The mixtures were either irradiated with red light (+hν) or kept in the dark (−hν). UV-vis absorbance at 351 nm was recorded after 20 and 40 min (**A**). HPLC profile of the 20 min irradiated sample containing SnPPA using a gradient of solution B (ACN) in solution A (TEAA buffer, 150 mM, 5% ACN, pH 7.4): From 0 to 10 min to 15% B until 15 min. From 15 to 20 min to 20% B until 35 min (**B**). All peaks were collected and analyzed by MALD-MS spectrometry (see Figure S2, SI). HPLC profile of 5′-phosphorylated DNA strand (**C**).

To investigate the cleavage of DNA **1** by photoreduction, we incubated DNA **1** with sodium ascorbate in the presence (“sample”) or absence (“blank”) of photosensitizer SnPPA. The photosensitizer was selected as a catalyst of photoreduction based on our previously published study (synthesis as described in [16], for MS spectra, see Figure S1, SI). The samples were either irradiated with red light (660 nm) (+hv) or kept in the dark (−hv), and the absorption at 351 nm (characteristic of the more abundant *trans* isomer of DNA **1**, see Figure 3B) was recorded after 20 min and 40 min (Figure 4A). In the presence of the photosensitizer and red light, the bleaching of the azobenzene takes place as detected by the reduced absorbance. In contrast, in the absence of either light or a catalyst, no degradation of DNA **1** is detectable. These data confirm the first step of the mechanism of DNA **1** photoreduction outlined in Scheme 2.

To identify the product(s) formed in the latter reaction, we subjected the sample after 40 min irradiation in the presence of SnPPA to HPLC. The corresponding profile (Figure 4B) shows one major peak (approximately 80% of eluted peaks after injection peak), that is eluted after 11.8 min. The corresponding MALDI-TOF-MS spectrum (Figure S3, SI) indeed shows the mass (calculated for C_49_H_66_N_11_O_35_P_5_ [M − H^+^]^−^: *m*/*z* = 1523 Da, found: *m*/*z* = 1524 Da) of the expected 5′-phosphorylated DNA after azobenzene reduction and subsequent 1,6-elimination of para-quinone methide followed by its quenching with water with the formation of 4-hydroxymethylaniline (Scheme 2). As an additional control, we synthesized the 5′-phosphorylated DNA strand and subjected it to HPLC (Figure 4C), showing the same elution time (11.8 min) as the product of the photoreduction, further confirming the identity of the cleavage product.

### 2.3. DNA ***1*** Cleavage Selectivity and RNA ***2*** Photoreduction

**Figure 5:**
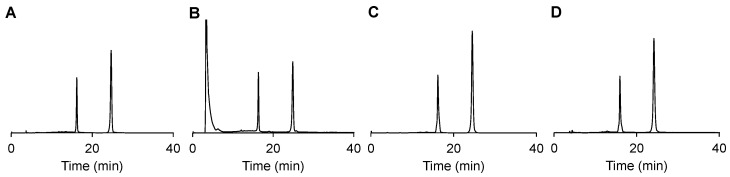
DNA **1** stability in PBS (10 mM, 150 mM NaCl pH 7.4) (**A**), in the presence of 200 mM H_2_O_2_ (**B**), KO_2_ (100 µM (1% DMSO)) (**C**), or NaSH (630 µM) (**D**) after 1 incubation at 37 °C as analyzed by HPLC using a gradient solution B (ACN) in solution A (TEAA buffer, 150 mM, 5% ACN, pH 7.4): From 0 to 10 min to 15% B until 15 min; from 15 to 20 min to 20% B until 35 min.

To challenge the selectivity of RF cleavage by red-light-induced electron transfer, DNA **1** was incubated with biorelevant redox species, namely, H_2_O_2_ (200 mM), KO_2_ (100 µM), and NaSH (630 µM), for 1 h. As shown in Figure 5A–D, no cleavage products could be detected under these conditions. Note that the large injection peak in B is caused by H_2_O_2_ light absorption. This proves the selectivity of RF cleavage by electron transfer, forming a solid foundation for applications in a biological context.

**Figure 6:**
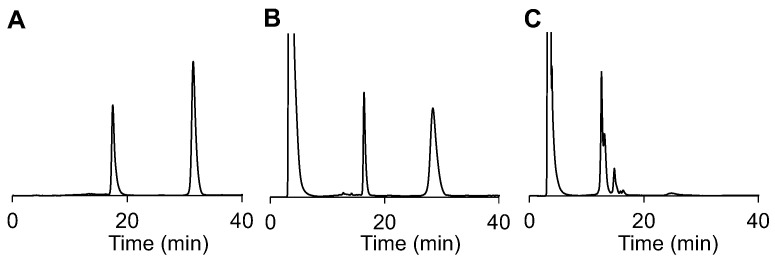
Photoreduction of RNA **2** in PBS buffer (10 mM, 150 mM NaCl, pH 7.4) containing sodium ascorbate (10 mM) and SnPPA (10 µM) as observed by HPLC. The sample was either directly injected into HPLC (**A**), incubated in the dark (**B**), or irradiated with red light for 30 min (**C**). A gradient of solution B (ACN) in solution A (TEAA buffer, 150 mM, 5% ACN, pH 7.4), from 0 to 10 min to 15% B until 20 min, then from 20 to 40 min to 30% B, was used. HPLC fractions were analyzed by MALDI-TOF-MS (see Figure S3, SI).

To approach our goal of in vitro application, we repeated the cleavage experiments on an RNA level by incubation of RNA **2** with sodium ascorbate and the photosensitizer SnPPA in the absence or presence of red light. In the dark, no cleavage takes place (compare Figure 6A,B). However, upon irradiation, two major cleavage products could be detected (Figure 6C). The first one, eluted after 12 min, corresponds to the phosphorylated RNA strand (calculated for C_200_H_250_N_69_O_151_P_21_ [M − H^+^]^−^: *m*/*z* = 6685 Da; found: *m*/*z* = 6676), according to the MALDI-TOF-MS spectra (Figure S3, SI). The second one, eluted 1 min later, corresponds to the thiophosphorylated RNA strand (calculated for C_200_H_250_N_69_O_150_P_21_S [M − H^+^]^−^: *m*/*z* = 6701 Da; found: *m*/*z* = 6703 Da). Both species would catalyze RNAi in a cellular context.

### 2.4. Knockdown Efficacy of RNA ***2/3***

**Figure 7:**
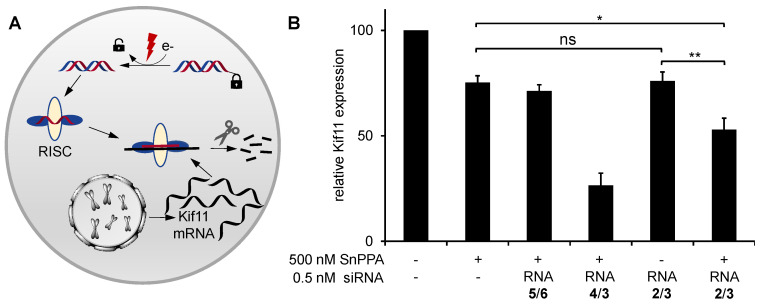
(**A**) After the photoreduction of the siRNA prodrug, the guide strand of the siRNA is uptaken into the RNA-induced silencing complex (RISC) to selectively cleave the complementary *KIF11* mRNA, resulting in reduced *KIF11* expression. (**B**) Relative *KIF11* mRNA concentrations of A2780 cells treated with 0.5 nM the unmodified *KIF11* siRNA **4**/**3** or the modified analog siRNA **2/3** for 38 h. As a negative control, siRNA **5/6**, which has no target mRNA in the cells, was applied. After 14 h incubation, SnPPA (500 nM, 0.1% DMSO, end concentration) was added to the indicated samples and incubated for 30 min. All samples were irradiated for 30 min with red light, and subsequently incubation was continued in the dark. As a housekeeping gene, GAPDH was used. 2^−ΔCt^ values were calculated and normalized to those of untreated cells. Three independent experiments were performed. Error bars indicate standard deviation. An unpaired Student’s *t*-test was performed for statistical analysis: * *p* < 0.05; ** *p* < 0.01; ns *p* ≥ 0.05.

Encouraged by these results, we annealed RNA **2** with its corresponding passenger strand RNA **3** and formulated it with the standard commercially available transfection agent Lipofectamine™ RNAiMAX for the transfection of A2780 cancer cells. As a target gene, we selected *KIF11*, a member of the kinesin-5 family, an essential enzyme for mitosis in most organisms. The inhibition of *KIF11* is reflected in the accumulation of cells in a G2 phase and an increase in the ratio of cell numbers in G2 and G1 states (G2/G1) [19]. We applied the unmodified *KIF11* siRNA **4**/**3** as a positive control and the scrambled siRNA **5**/**6** as a negative control. Upon completed transfection (14 h), we added the photosensitizer SnPPA. Then, 30 min later, all samples were irradiated with red light (660 nm) for 30 min. After an additional 23 h, the relative *KIF11* expression was quantified by RT-qPCR. The overall incubation time of the cells with the prodrug and controls was 38 h. It is expected that, upon light irradiation, the azobenzyl group at the 5′ end of the antisense strand is cleaved, allowing the siRNA to be activated by the RNA-induced silencing complex (RISC) (Figure 7A). The siRNA antisense strand then serves as a template for the selective binding of mRNAs with the corresponding sequence (in this case: *KIF11*), followed by mRNA degradation. By RT-qPCR, the relative mRNA content of single genes can be quantified and normalized to that of a consecutively expressed housekeeping gene (in this case: GAPDH). As shown in Figure 7B, RNA **2**/**3**, indeed, significantly decreased the *KIF11* mRNA concentration in the presence of the photosensitizer as compared to cells only treated with the photosensitizer (*p* < 0.01, unpaired Student’s *t* test). In contrast, no significant RNAi could be observed in the absence of SnPPA. These results not only demonstrate the successful activation of RNA **2**/**3** in cells but also its selective on-demand activation in the presence of the photosensitizer SnPPA.

## 3. Materials and Methods

### 3.1. Synthesis of Modified Oligonucleotides

Automated oligonucleotide synthesis was performed on a 1 μmol scale by using the standard (3′→5′) synthesis according to the recommendations of the manufacturer. For DNA synthesis, dC(bz) CPG (1000 Å, 28 µmol/g) was used, and for RNA synthesis, dT CPG (1000 Å, 25–35 µmol/g) was used. The phosphoramidite-carrying RF was coupled manually under argon atmosphere for 10 min. For this, the phosphoramidite in water-free acetonitrile (0.1 mL, 0.1 M) was mixed in the solid phase with a solution of ETT activator in acetonitrile (0.1 mL, 0.5 M). After oxidation with a standard iodine solution and DMT-deprotection, coupling was repeated. For RNA **2**, oxidation was performed with 3-ethoxy-1,2,4-thiazoline-5-one (EDITH, 0.05 M, in acetonitrile) with 20 s oxidation time. The synthesized and chemically modified oligonucleotides were purified on reversed-phase HPLC with a gradient of solution B (ACN) in solution A (TEAA buffer, 150 mM, 5% ACN, pH 7.4). For DNA, a gradient from 0 to 10 min to 15% B until 15 min and then from 15 to 20 min to 20% B until 35 min was used. RNA was purified with a gradient from 0 to 10 min to 15% B until 20 min, then from 20 to 40 min to 30% B. Concentrations of modified and non-modified RNAs and DNAs were determined by measuring the absorption at 260 nm. Annealing of RNA/DNA strands (10 µM each strand) was performed in Tris buffer (100 mM Tris, pH 7.8, 500 mM NaCl, and 10 mM EDTA) by heating the mixture to 90 °C for 15 min and cooling it by 3 °C per 2 min to 22 °C.

### 3.2. Cis-Trans Isomerization of DNA ***1***

UV-vis absorbance of DNA **1** (15 µM) in PBS (10 mM, 150 mM NaCl, pH 7.4) was measured before (t = 0 min) and after irradiation with 365 nm light for 1 min (Figure 3B). In addition, DNA **1** (20 µM) in PBS (10 mM, 150 mM NaCl, pH 7.4) was injected into HPLC before (Figure 2C) and after 5 min irradiation at 365 nm (Figure 3D) and after 5 min irradiation with 365 nm plus an additional 5 min incubation (Figure 3E) using a gradient of solution B (ACN) in solution A (TEAA buffer, 150 mM, 5% ACN, pH 7.4): From 0 to 10 min to 15% B until 15 min. From 15 to 20 min to 20% B until 35 min.

### 3.3. DNA ***1*** Cleavage by Red-Light-Induced Photoreduction

DNA **1** (50 µM) was dissolved in PBS (10 mM, 150 mM NaCl, 10 mM sodium ascorbate, pH 7.4) in the presence or absence of SnPPA (4 µM) (for synthesis and structure see [16]). The mixtures were either irradiated (+hν) or kept in the dark (−hν) and UV-Vis spectra were recorded after 20 and 40 min (351 nm) (Figure 4A). The 20 min irradiated sample containing SnPPA was additionally injected into HPLC using a gradient of solution B (ACN) in solution A (TEAA buffer, 150 mM, 5% ACN, pH 7.4): From 0 to 10 min to 15% B until 15 min. From 15 to 20 min to 20% B until 35 min (Figure 4B). All peaks were collected and analyzed by MALD-MS spectrometry (see SI, Figure S2). The HPLC profile was compared to that of the unmodified DNA strand (Figure 4C).

### 3.4. Stability in Presence of Oxidizing/Reducing Agents

DNA **1** (75 µM) was dissolved in PBS (10 mM, 150 mM NaCl pH 7.4) (Figure 5A) in presence of 200 mM H_2_O_2_ (Figure 5B), in presence of KO_2_ (100 µM (1% DMSO)) (Figure 5C), or in presence of NaSH (630 µM) (Figure 5D) and incubated for 1 h at 37 °C. Solutions were injected to reversed-phase HPLC using a gradient solution B (ACN) in solution A (TEAA buffer, 150 mM, 5% ACN, pH 7.4): From 0 to 10 min to 15% B until 15 min. From 15 to 20 min to 20% B until 35 min.

### 3.5. RNA ***2*** Cleavage by Red-Light-Induced Photoreduction

RNA **2** (50 µM) was dissolved in PBS buffer (10 mM, 150 mM NaCl, pH 7.4) containing sodium ascorbate (10 mM) and SnPPA (10 µM) and directly injected into HPLC (Figure 6A) or kept in the dark (−hν) (Figure 6B) or irradiated with red light for 30 min at 23 °C (Figure 6C) and subsequently injected into HPLC using a gradient solution B (ACN) in solution A (TEAA buffer, 150 mM, 5% ACN, pH 7.4): from 0 to 10 min to 15% B until 20 min, then from 20 to 40 min to 30% B. HPLC fractions were analyzed by MALDI-TOF-MS (see Figure S3, SI)

### 3.6. Transfection

A2780 were seeded in 6-well plates (100 cells/μL, 2 mL RPMI 1640, containing FBS (5%), penicillin/streptomycin (1%), and L-Glutamine (1%) per well). For the transfection, 1 pmol (for 0.5 nM end concentration) siRNA in biograde water (10 µL) was diluted with Gibco™ Opti-MEM™ Reduced Serum Media (Opti-MEM) (40 μL). In addition, Lipofectamine^TM^ RNAiMAX (1.5 μL) was diluted in Opti-MEM (48.5 μL) and incubated for 5 min at 22 °C before both solutions were combined. The transfection solution was then incubated for 20 min at 23 °C with recurring shaking every 5 min. The cells were washed with PBS (2.5 mL per well) and a fresh portion of RPMI 1640 medium (1.9 mL per well) containing FBS (5%), penicillin/streptomycin (1%), and L-Glutamine (1%) was added. Finally, the 100 μL transfection solution was added to the cells. The samples were incubated for 14 h.

### 3.7. Relative Gene Expression Quantification by RT-qPCR

SnPPA (500 nM, 0.1% DMSO) was added to the cells. As a control, to one of the siRNA **2**/**3**-treated sample, no SnPPA was added. The samples were incubated for 30 min in the dark, and subsequently irradiated for 30 min with a red LED lamp consisting of eighteen 2-watt super-bright red LEDs (λ = 660 nm, Flux = 1086 lm, efficacy: 56.6 lm/w; half width at half maximum = 26.4 nm; distance to samples = 30 cm). The samples were incubated for 23 h in the dark. To isolate total RNA from cells, the cultivation medium was removed, the cells were washed with PBS, and TRI reagent^®^ (1 mL/well) was added for lysis. After 5 min incubation at 22 °C, the cell lysate was transferred into 1.5 mL Eppendorf tubes, and the aqueous phase of the samples was extracted by adding chloroform (200 µL, 4 °C) followed by thorough mixing and centrifugation (12,000× *g*, 4 °C, 15 min). The aqueous phase was mixed with ethanol (400 µL, 4 °C) and transferred onto Zymo-Spin IC Columns. The samples were subsequently centrifuged (1 min, 8000× *g*, 4 °C). The eluate was discarded. The RNA on the column was washed first with sodium acetate buffer (3 M, 500 µL, 4 °C, pH = 5.2) and secondly with ethanol (500 µL, 75%, *v*/*v*, in water, 4 °C), in three steps: solvent addition, subsequent centrifugation as above, and eluate removal. Finally, pure ethanol (500 µL, 4 °C) was added and the samples were centrifuged (3 min, 10,000× *g*, 4 °C) to dry the column. The RNA was eluted by adding RNase-free water (25 µL), followed by incubation for 5 min at 22 °C, and subsequent centrifugation (2 min, 8000× *g*, 4 °C). To transcribe the isolated RNA into cDNA, RNA (1 µg in 12.5 µL RNase-free water) was incubated (65 °C for 5 min) with random hexamer primer (1 µL). Subsequently, reverse transcriptase (0.5 µL), RT 5x buffer (4 µL), and dNTPs (2 µL) were added per sample. Finally, the reverse-transcription protocol (10 min at 25 °C; 60 min at 42 °C; 10 min at 70 °C; forever at 4 °C) was run at the Dual Block Gradient PCR Thermal Cycler. For the relative quantification of the genes of interest, LightCycler^®^ 480 SYBR^®^ Green Master (5 µL) (Roche Diagnostics Deutschland GmbH, Mannheim, Germany) was mixed with a primer pair (1 µL) and cDNA (4 µL, 1:10 diluted) and subjected to the LightCycler^®^ 480 (Roche Diagnostics Deutschland GmbH, Mannheim, Germany). The applied primer sequences were CAGCTGAAAAGGAAACAGCC; ATGAACAATCCACACCAGCA for *KIF11* and CTTCACCACCATGGAGGAGGC; GGCATGGACTGTGGTCATGAG for the housekeeping gene GAPDH. The reaction mixtures were initially heated at 95 °C for 5 min, followed by 45 cycles, which consisted of 10 s at 95 °C, 30 s 60 °C, and 10 s at 72 °C. The Ct value of the *KIF11* cDNA amplification of each sample was subtracted by the Ct value of the GAPDH cDNA amplification of the same sample (∆Ct). For the evaluation of the fold change, the 2^−ΔCt^ value was calculated. The 2^−ΔCt^ value of untreated cells was set to 100. Three independent experiments were performed and the mean ± standard deviation were calculated and are shown in Figure 7.

### 3.8. Statistical Analysis of Data

For statistical analysis of data, an unpaired Student’s *t*-test was performed using GraphPad Prism Software: * *p* < 0.05; ** *p* < 0.01. Difference between experimental average values, for which *p* ≥ 0.05, were considered to be statistically not significant (ns).

## 4. Conclusions

In this study, we present electron-rich azobenzene “RF” as a suitable promoiety for the design of siRNAs activated under reductive conditions upon irradiation with red light. This can be achieved in the presence of SnPPA acting as a photocatalyst mediating the transfer of electrons from intracellular electron sources (e.g., ascorbate) to the azobenzene. In particular, the RF-based prodrug is not reduced either in the dark or upon irradiation with red light in the presence of bioavailable redox species such as H_2_O_2_, NaSH, KO_2_, or ascorbate if SnPPA is not present. We first characterized the promoiety on a DNA model strand in cell-free settings and then successfully synthesized and characterized an RF-carrying RNA strand. Finally, we transferred the concept into cells, by application of an RF-siRNA prodrug, which was transfected with a standard transfection agent into ovarian carcinoma cells (A2780). We also showed that, in cells, the siRNA is selectively activated upon red light irradiation in the presence of the photosensitizer SnPPA inducing *KIF11* knockdown. With this siRNA prodrug approach, we avoid the formation of toxic metabolites such as ^1^O_2_ and anthraquinone, making this concept superior to the previously published one. The RNAi is triggered by biologically tolerated red light, due to the application of the catalyst SnPPA, which uses naturally abundant metabolites, such as ascorbic acid, as electron sources. Presumably, the promoiety described here can be easily attached to any siRNA sequence by the presented synthesis route and the resulting prodrug can be formulated with standard transfection reagents, making this approach a versatile tool to improve siRNA selectivity.

## Data Availability

Data is contained within the article and the Supplementary Material.

## Supplementary Materials

The following supporting information can be downloaded at https://www.mdpi.com/article/10.3390/molecules28104204/s1, with details about applied chemicals and instruments, Scheme S1: Structure of DMT-RF-carrying phosphoramidite, Scheme S2: Structure of DNA **1**, Scheme S3: Structure of RNA **2**, Figure S1: High-resolution mass spectrum of SnPPA, Figure S2: MALDI-TOF-MS spectra of DNA **1** stability test, Figure S3: MALDI-TOF-MS spectra of DNA **1** cleavage experiments, details about cells and cell culture.

## References

[1] Scott L.J. (2020). Givosiran: First Approval. Drugs.

[2] Scott L.J., Keam S.J. (2021). Lumasiran: First Approval. Drugs.

[3] Wood H. (2018). FDA approves patisiran to treat hereditary transthyretin amyloidosis. Nat. Rev. Neurol..

[4] Lamb Y.N. (2021). Inclisiran: First Approval. Drugs.

[5] Keam S.J. (2022). Vutrisiran: First Approval. Drugs.

[6] Han S.P., Scherer L., Gethers M., Salvador A.M., Salah M.B.H., Mancusi R., Sagar S., Hu R., DeRogatis J., Kuo Y.H. (2022). Programmable siRNA pro-drugs that activate RNAi activity in response to specific cellular RNA biomarkers. Mol. Ther. Nucleic Acids.

[7] Hayashi J., Nishigaki M., Ochi Y., Wada S.I., Wada F., Nakagawa O., Obika S., Harada-Shiba M., Urata H. (2018). Effective gene silencing activity of prodrug-type 2′-O-methyldithiomethyl siRNA compared with non-prodrug-type 2′-O-methyl siRNA. Bioorg. Med. Chem. Lett..

[8] Mikat V., Heckel A. (2007). Light-dependent RNA interference with nucleobase-caged siRNAs. RNA.

[9] Rühle J., Klemt I., Abakumova T., Sergeeva O., Vetosheva P., Zatsepin T., Mokhir A. (2022). Reactive oxygen species-responsive RNA interference. Chem. Commun..

[10] Antunes F., Cadenas E. (2000). Estimation of H_2_O_2_ gradients across biomembranes. FEBS Lett..

[11] Rybak C.J., Andjaba J.M., Fan C., Zeller M., Uyeda C. (2023). Dinickel-Catalyzed N=N Bond Rotation. Inorg. Chem..

[12] Johnson T.G., Sadeghi-Kelishadi A., Langton M.J. (2022). A Photo-responsive Transmembrane Anion Transporter Relay. J. Am. Chem. Soc..

[13] Samanta S., Beharry A.A., Sadovski O., McCormick T.M., Babalhavaeji A., Tropepe V., Woolley G.A. (2013). Photoswitching azo compounds in vivo with red light. J. Am. Chem. Soc..

[14] Tian X., Li Z., Sun Y., Wang P., Ma H. (2018). Near-Infrared Fluorescent Probes for Hypoxia Detection via Joint Regulated Enzymes: Design, Synthesis, and Application in Living Cells and Mice. Anal. Chem..

[15] Guisán-Ceinos S., Rivero A.R., Romeo-Gella F., Simón-Fuente S., Gómez-Pastor S., Calvo N., Orrego A.H., Guisán J.M., Corral I., Sanz-Rodriguez F. (2022). Turn-on Fluorescent Biosensors for Imaging Hypoxia-like Conditions in Living Cells. J. Am. Chem. Soc..

[16] Dutta S., Rühle J., Schikora M., Deussner-Helfmann N., Heilemann M., Zatsepin T., Duchstein P., Zahn D., Knör G., Mokhir A. (2020). Red light-triggered photoreduction on a nucleic acid template. Chem. Commun..

[17] Jiang M., Zhuang H., Xia R., Gan L., Wu Y., Ma J., Sun Y., Zhuang Z. (2017). KIF11 is required for proliferation and self-renewal of docetaxel resistant triple negative breast cancer cells. Oncotarget.

[18] Kinesin Family Member 11 [Homo sapiens (Human)]. https://www.ncbi.nlm.nih.gov/gene/3832.

[19] Meyer A., Mokhir A. (2014). RNA Interference Controlled by Light of Variable Wavelength. Angew. Chem. Int. Ed..

