# Supplementary material for: Triggering RNA Interference by Photoreduction under Red Light Irradiation
Source: Molecules. 2023 May 20;28(10):4204. doi: 10.3390/molecules28104204 (PMC10223155; doi:10.3390/molecules28104204)
Supplement: Supplementary file 1 [file molecules-28-04204-s001.zip › molecules-2398416-supplementary.pdf]

Supporting Information

Triggering RNA Interference by Photoreduction under Red Light Irradiation

Jennifer Rühle, Insa Klemt, and Andriy Mokhir

Table of content

S1. Synthesis page S1

S2. Tests of conjugates page S3

S3. Cell and cell culture page S5

S1. Synthesis

S1.1. Chemicals

If not stated otherwise, all chemicals were acquired commercially from Sigma-Aldrich (Germany) in at least 98 percent purity. The reagents were used without further purification. Anhydrous solvents and NMR solvents were stored under nitrogen or argon atmosphere and, if possible, with the use of a molecular sieve. As the solid phase for DNA synthesis, dC(bz) CPG (1000 Å, 28 µmol/g, Sigma Aldrich) was used, and for RNA synthesis, dT CPG (1000 Å, 25 – 35 µmol/g, Sigma Aldrich) was used. The strands were synthesized with DMT-dT and DMT-2´-O-TC (rA(bz), rC(ac), rU, and rG(ib)) phosphoramidites purchased from Sigma Aldrich. Gibco™ Opti-MEM™ Reduced Serum Media (Opti-MEM), Lipofectamine® RNAiMAX, random hexamer primer and dNTPs, the reverse transcriptase, and its 5x buffer were all obtained from Thermo Fisher Scientific, Germany. The Zymo-Spin IC Columns were purchased from Zymo Research Corporation, US. LightCycler® 480 SYBR® Green Master from Roche Diagnostics GmbH (Germany) was used for the qPCR analysis. For the microscopy, 35 mm µ-imaging dishes (ibidi GmbH, Germany) were used

S1.2. Instruments

Automated oligonucleotide synthesis was performed on an H-8 synthesizer (K&A Laborgeräte GbR, Germany). Purification of oligonucleotide conjugates was performed on a Prominence UFPLC (Shimadzu) equipped with an EC 250/4.6 Nucleosil 100-10 C18 column (Macherey-Nagel, Germany). The chromatographic separations were performed at 22 °C. HPLC fractions were analyzed on an UltrafleXtreame MALDI-TOF mass spectrometer (Bruker). Samples were prepared by dried droplet method on an MTP 384 polished steel plate (Bruker). Concentrations of modified and non-modified RNAs and DNAs were determined with a NanoDropTM UV-visible spectrophotometer (Thermo

Fisher Scientific, Germany). For irradiation, a red LED lamp consisting of eighteen 2-watt super bright red LEDs (λ = 660 nm, Flux = 1086 lm, efficacy: 56.6 lm/w; half width at half maximum = 26.4 nm) with 30 cm distance to the sample was used. The reverse transcription was performed with a Dual Block Gradient PCR Thermal Cycler GE4852T™ (Biogen, US). For the qPCR experiments, the LightCycler® 480 from Roche Molecular Systems, Inc. (US) was used.

S1.3. Synthesis of phosphoramidite compound and modified oligonucleotides

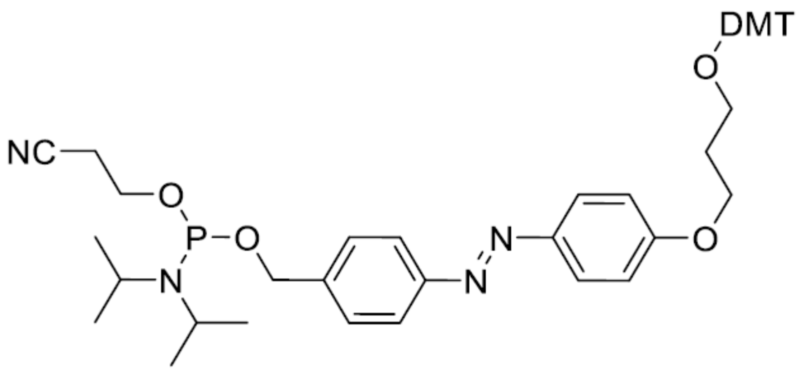

Scheme **S1**: Structure of DMT-RF-carrying phosphoramidite used for DNA/RNA synthesis.

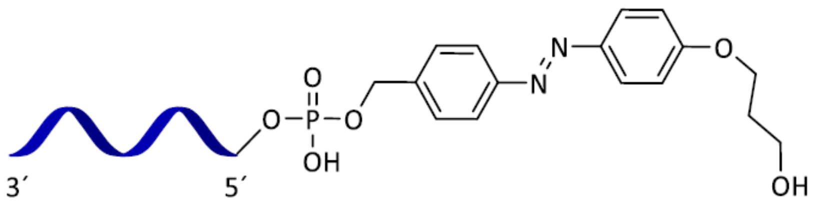

Scheme S2. Structure of DNA **1.**

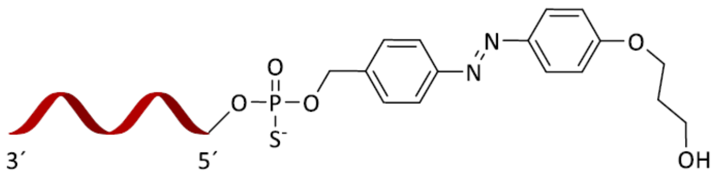

Scheme S3. Structure of RNA **2**.

S2. Tests of conjugates

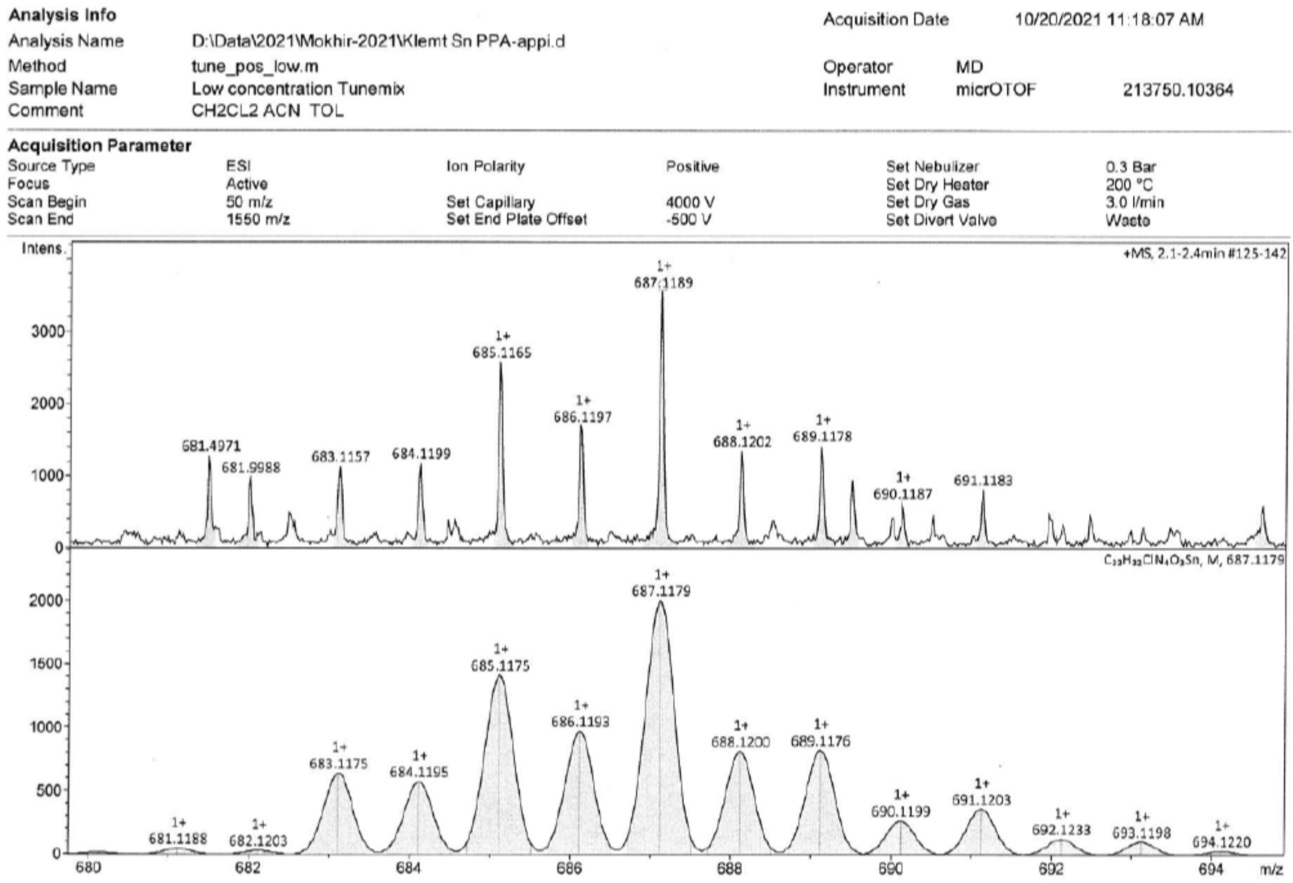

Figure S1. High-resolution mass spectrum of [Sn(P~OH)Cl2]*EtOH.

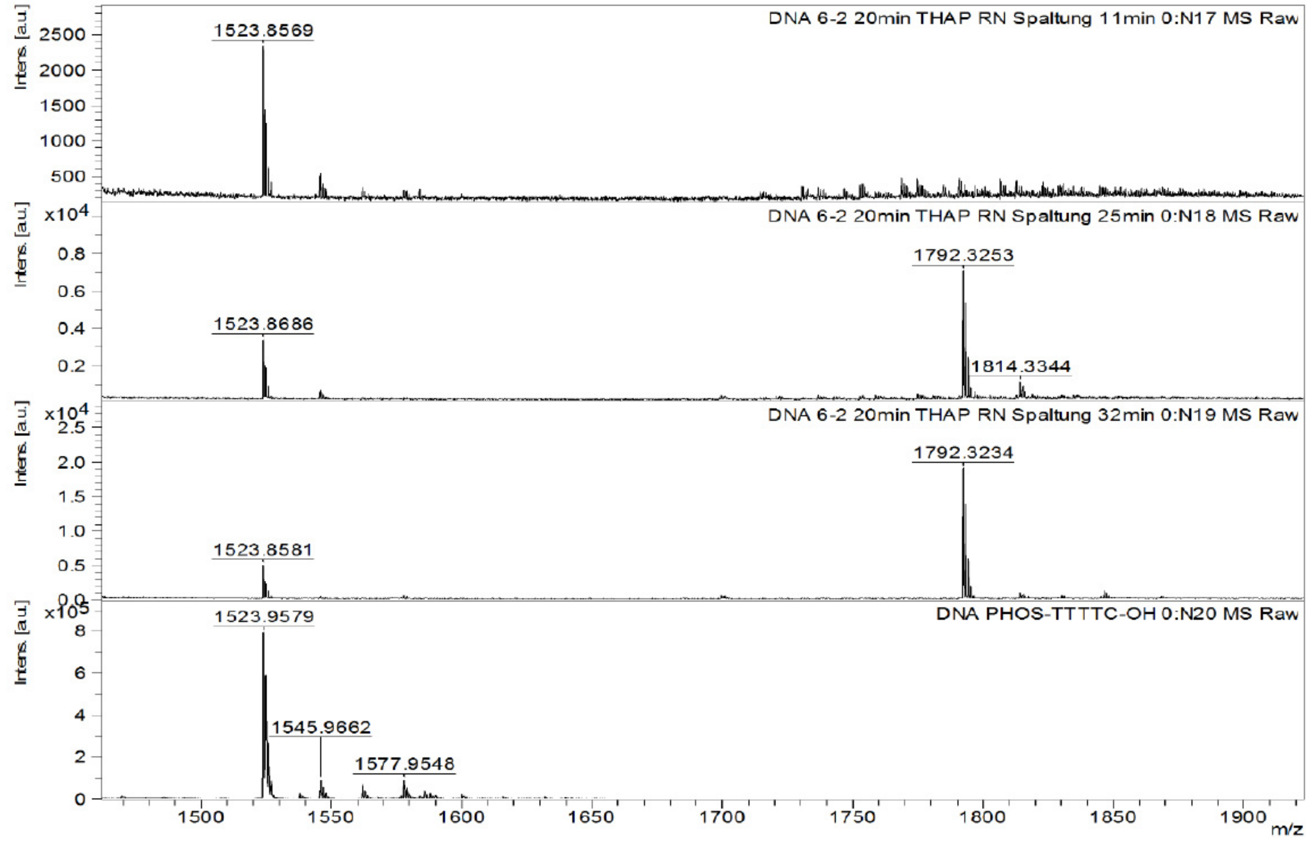

Figure S2. Incubation of DNA **1** with sodium ascorbate in the presence of SnPPA (Figure 4B). Upper spectrum of cleavage product at 11 min. Both middle spectra correspond to the peaks of residual DNA **1** at 25 min and 32 min which was not cleaved (calculated for $C_{65}H_{82}N_{13}O_{37}P_5$ $[M - H^+]^-$: m/z = 1791 Da; found: $[M - H^+]^-$: *m/z* = 1792 Da). (Figure 4B) and lowest spectrum of the reference (phosphorylated strand, Phos-TTTTC, Figure 4C). Expected mass: calculated for $C_{49}H_{66}N_{11}O_{35}P_5$ $[M - H^+]^-$: m/z = 1523 Da.

A

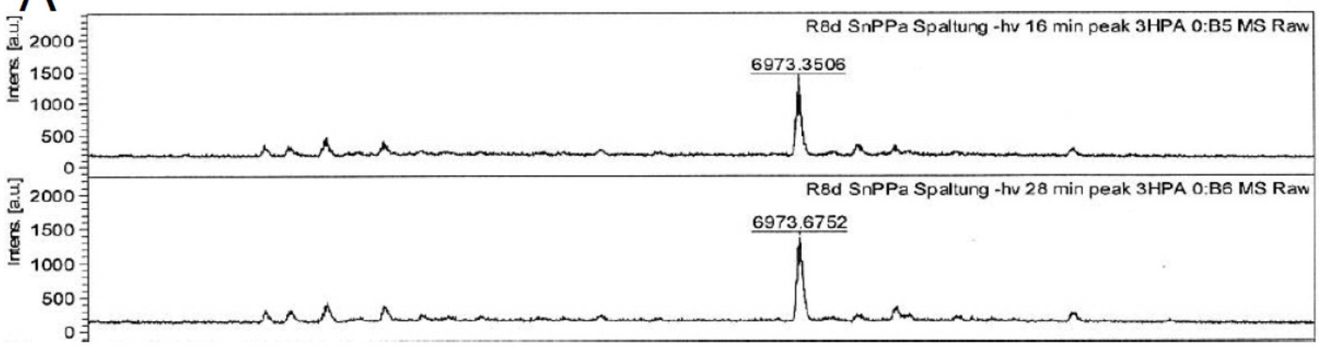

B

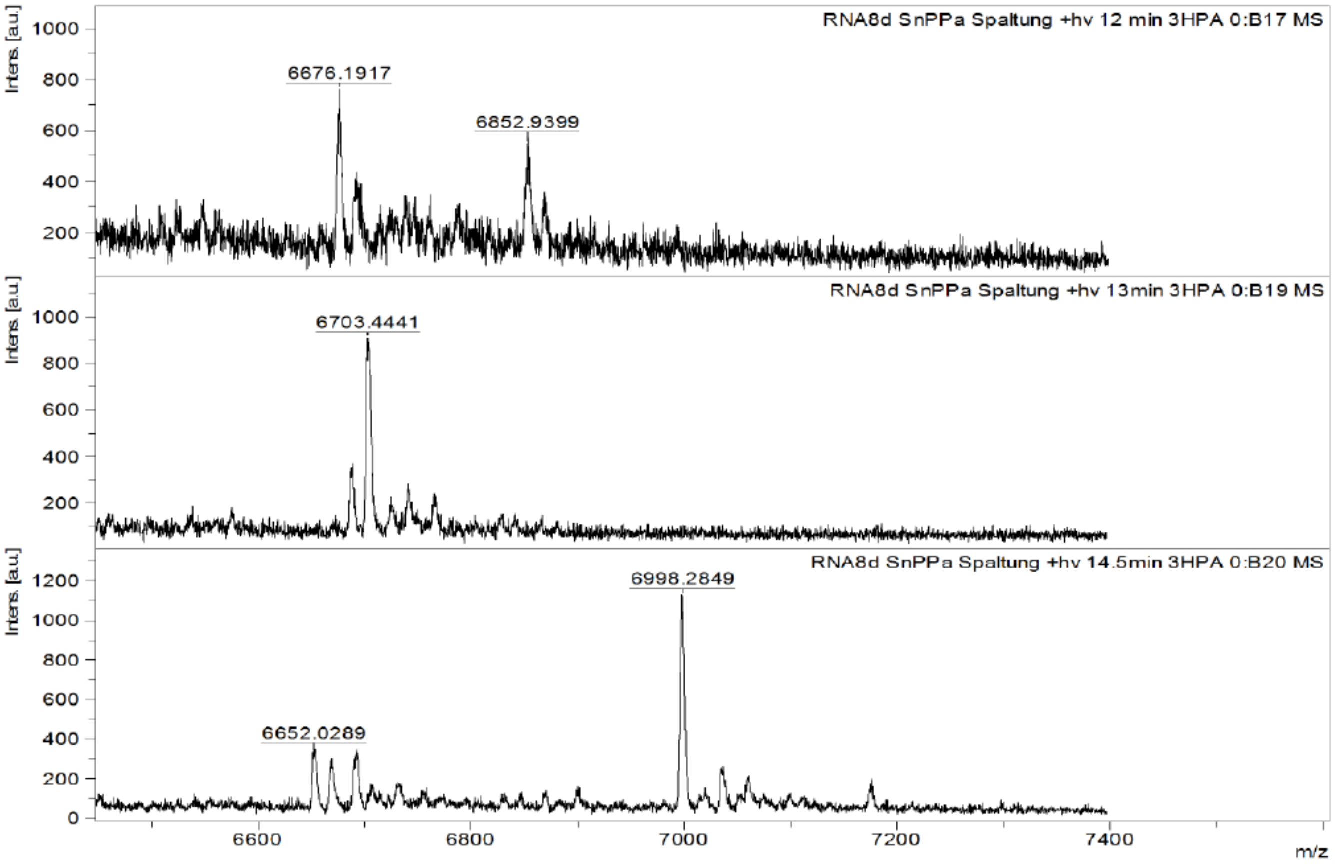

Figure S3: MALDI-TOF masses for cleavage of RNA **2** with SnPPa (for HPLC traces and details see Figure 6). **A**: Sample without irradiation (-hv) for both isomeric peaks (elution after 16 min and 28 min, respectively) of RNA **2** (calculated for $C_{216}H_{266}N_{71}O_{152}P_{21}S$ $[M - H^+]^-$: *m/z* = 6970 Da). **B**: Sample with irradiation (+hv): 12 min peak (5´-phos-antisense strand, calculated for $C_{200}H_{250}N_{69}O_{151}P_{21}$ $[M - H^+]^-$: *m/z* = 6685 Da), 13 min peak (5´- thiophos-antisense strand, calculated for $C_{200}H_{250}N_{69}O_{150}P_{21}S$ $[M - H^+]^-$: *m/z* = 6701 Da)), 14.5 min peak (product unknown).

S3. Cells and cell culture

Human ovarian cancer cell line A2780 was purchased from Sigma-Aldrich and cultivated in Roswell Park Memorial Institute (RPMI) 1640 medium supplemented with fetal bovine serum (FBS, 10%), penicillin/streptomycin (1%), and L-Glutamine (1%). Cells were cultivated to around 80 % confluence at 37 °C with 5% CO2.
